# Operation of a MOEMS Deformable Mirror in Cryo: Challenges and Results

**DOI:** 10.3390/mi8080233

**Published:** 2017-07-27

**Authors:** Frederic Zamkotsian, Patrick Lanzoni, Rudy Barette, Michael Helmbrecht, Franck Marchis, Alex Teichman

**Affiliations:** 1 Aix Marseille Univ, CNRS, LAM, Laboratoire d’Astrophysique de Marseille, 38 rue Frederic Joliot Curie, 13388 Marseille CEDEX 13, France; patrick.lanzoni@lam.fr (P.L.); rudy.barette@lam.fr (R.B.); 2 Iris AO, 2930 Shattuck Avenue #304, Berkeley, CA 94705, USA; info@irisao.com or michael.helmbrecht@irisao.com (M.H.); alex.teichman@irisao.com (A.T.); 3 Carl Sagan Center, SETI Institute, 189 Bernardo Ave, Mountain View, CA 94043, USA; fmarchis@seti.org

**Keywords:** MEMS mirror arrays, MOEMS, cryogenic testing, adaptive optics, wavefront correction

## Abstract

Micro-opto-electro-mechanical systems (MOEMS) Deformable Mirrors (DM) are key components for next generation optical instruments implementing innovative adaptive optics systems, both in existing telescopes and in the future ELTs. Characterizing these components well is critical for next generation instruments. This is done by interferometry, including surface quality measurement in static and dynamical modes, at ambient and in vacuum/cryo. We use a compact cryo-vacuum chamber designed for reaching 10–6 mbar and 160 K in front of our custom Michelson interferometer, which is able to measure performance of the DM at actuator/segment level and at the entire mirror level, with a lateral resolution of 2 µm and a sub-nanometer z-resolution. We tested the PTT 111 DM from Iris AO: an array of single crystalline silicon hexagonal mirrors with a pitch of 606 µm, able to move in tip, tilt, and piston (stroke 5–7 µm, tilt ±5 mrad). The device could be operated successfully from ambient to 160 K. An additional, mainly focus-like, 500 nm deformation of the entire mirror is measured at 160 K; we were able to recover the best flat in cryo by correcting the focus and local tip-tilts on all segments, reaching 12 nm rms. Finally, the goal of these studies is to test DMs in cryo and vacuum conditions as well as to improve their architecture for stable operation in harsh environments.

## 1. Introduction

Wavefront correction is a key issue in a wide range of applications, from physics to biology or astronomy. Collimating and focusing with high accuracy a very large number of photons in high energy lasers, correcting the wavefront through diffuse or inhomogeneous media for sharp retinal and tissue imaging, or correcting, in closed loop, the atmospheric turbulence for revealing the faintest or most remote objects in the Universe, requires high performance wavefront correction systems.

**Figure 1:**
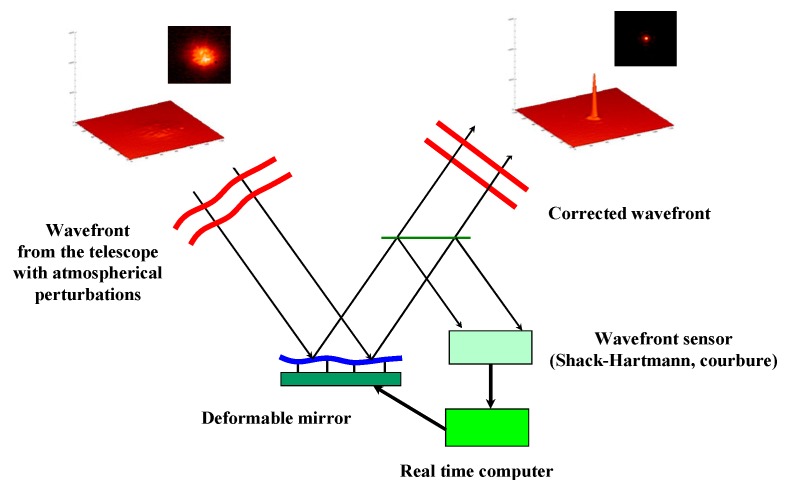
Schematic of a wavefront correction system.

In astronomy, high performance adaptive optical (AO) systems are being studied around the world for next generation instrumentation of 10 m-class telescopes as well as for future extremely large optical telescopes. Adaptive optics systems are based on a combination of three elements: the wavefront sensor for measuring the shape of the wavefront arriving in the telescope; the deformable mirror for correcting the wavefront; and, finally, the real time computer for closing the loop of the system at a frequency ranging from 0.5 to 3 kHz, in order to follow the evolution of the atmospherical perturbations (Figure 1). 

Four main types of AO systems have been built or are under development: Single-Conjugate Adaptive Optics (SCAO), Multi-Conjugate Adaptive Optics (MCAO), Multi-Object Adaptive Optics (MOAO), and Extreme Adaptive Optics (ExAO). These AO systems are associated with different types of WaveFront Sensors (WFS), combined with natural guide stars or laser guide stars, and different architectures of Deformable Mirrors (DM). Numerous science cases will use these AO systems: SCAO, the “classical” AO system, will provide accurate narrow field imagery and spectroscopy; MCAO, wide field imagery and spectroscopy; MOAO, distributed partial correction AO, and high dynamic range AO for the detection and the study of circumstellar disks and extra-solar planets. Corrected fields will vary from few arcsec to several arcmin. 

These systems require a large variety of deformable mirrors with very challenging parameters. For a 8 m telescope, the number of actuators varies from a few 10 up to 5000; these numbers increase impressively for a 40 m telescope, ranging from a few 100 to over 50,000 (the inter-actuator spacing from less than 200 µm to 1 mm, and the deformable mirror size from 10 mm to a few 100 mm). Conventional technology cannot provide this wide range of deformable mirrors. The development of new technologies based on micro-opto-electro-mechanical systems (MOEMS) is promising for future deformable mirrors. The major advantages of the micro-deformable mirrors (MDM) are their compactness, scalability, and specific task customization using elementary building blocks. This technology permits the development of a complete generation of new mirrors. However this technology also has some limitations. For example, pupil diameter is an overall parameter and for a 40 m primary telescope, the internal pupil diameter cannot be reduced below 0.5 m. According to the maximal size of the wafers (8 inches), a deformable mirror based on MOEMS technology cannot be built into one piece. New AO architectures have been proposed to avoid this limitation [1].

This new family of deformable mirrors will have also to fulfill some additional requirements: their ability to work in vacuum and at cryogenic temperatures. In order to study the early Universe, astronomers need to image and characterize astronomical objects (galaxies, quasars, large scale structures, etc.) in the infra-red part of the electromagnetic spectrum. At these wavelengths, the background noise of the instruments themselves must be reduced drastically by cooling them down at cryogenic temperatures, the longer the wavelength, the lower the temperature. Another challenge is to introduce MOEMS devices in future space instruments for reaching unprecedented performance. For all these applications, designing, testing, and operating MOEMS deformable mirrors in harsh environments (vacuums, cryogenic temperatures) is a critical issue.

For several years Laboratoire d’Astrophysique de Marseille (LAM) has been involved in the conception of new MOEMS devices as well as in the characterization of these components for the future instrumentation of ground-based and space telescopes. These studies include programmable slits for application in multi-object spectroscopy (JWST, European networks, EUCLID, BATMAN), deformable mirrors for adaptive optics, and programmable gratings for spectral tailoring.

We are particularly engaged in a European development of micromirror arrays (MMA) called MIRA for generating reflective slit masks in future Multi-object spectroscopy (MOS) instruments; this technique is a powerful tool for space and ground-based telescopes for the study of the formation and evolution of galaxies. MMA with 100 × 200 µm^2^ single-crystal silicon micromirrors were successfully designed, fabricated, and tested. Arrays are composed of 2048 micromirrors (32 × 64) with a peak-to-valley deformation less than 10 nm, and a tilt angle of 24° for an actuation voltage of 130 V. The micromirrors were actuated successfully before, during, and after cryogenic cooling, down to 162 K. The micromirror surface deformation was measured at cryo and is below 30 nm peak-to-valley [2,3]. In order to fill large focal planes (mosaicing of several chips), we are currently developing large micromirror arrays integrated with their electronics.

LAM is also leading the conception and realization of new MOEMS-based instruments, including the development of the Digital-Micromirror-Device (DMD)-based MOS instrument, to be mounted on the Telescopio Nazionale Galileo (TNG) by mid-2018 and called BATMAN [4].

In this paper, we present the specific set-up for the interferometric characterization of a segmented deformable mirror from Iris AO, in vacuum and at cryogenic temperatures. The results on the mirror surface operated from ambient down to 160 K are shown for the first time and analyzed.

## 2. Deformable Mirrors

Three main Micro-Deformable Mirrors (MDM) architectures are under study in different laboratories and companies. First, the bulk micro-machined continuous-membrane deformable mirror, studied by Delft University and OKO Company (Rijswijk, The Netherlands), is a combination of bulk silicon micromachining with standard electronics technology [5]. This mirror is formed by a thin flexible conducting membrane, coated with a reflective material, and stretched over an electrostatic electrode structure. This mirror shows very good mirror quality, but the mean deformed surface is a concave surface, and the number of actuators cannot be scalable to hundreds of electrodes. Second, the segmented, micro-electro-mechanical deformable mirror realized by Iris AO [6] consists of a set of segmented piston-tip-tilt moving surfaces, fabricated in dense array. Third, the surface micro-machined continuous-membrane deformable mirror made by Boston Micromachines Corporation (BMC) is based on a single compliant optical membrane supported by multiple attachments to an underlying array of surface-normal electrostatic actuators [7]. This device has been demonstrated recently in several AO systems, including the Gemini Planet Imager (GPI) instrument on Gemini telescope. The third concept shows limited strokes for large driving voltages, and the mirror surface quality may need further improvement for Extreme AO. All of these devices are based on silicon or polysilicon materials.

Two candidates, BMC deformable mirrors and Iris AO deformable mirrors, are foreseen for characterization in vacuum and at cryogenic temperatures, but none has yet been fully operated at cryogenic temperatures. Their architectures are presented in this paragraph.

### 2.1. BMC Deformable Mirror

**Figure 2:**
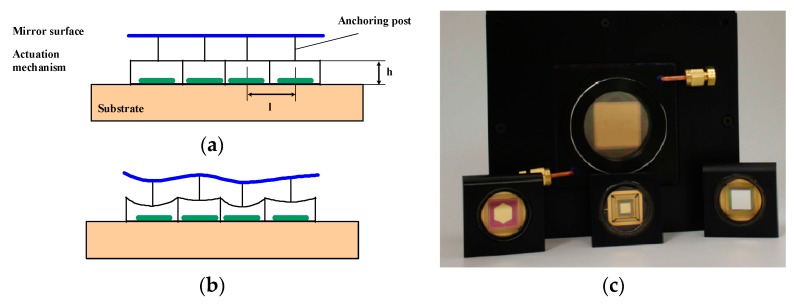
Continuous membrane Micro-Deformable Mirrors (MDM) from Boston Micromachines Corporation (BMC): schematic view of the (**a**) MDM non-actuated, (**b**) MDM actuated; (**c**) devices picture (courtesy from BMC).

BMC produces advanced MEMS deformable mirrors. The concept is based on an array of electrostatic actuators linked one by one to a continuous top mirror surface with etch holes (Figure 2). Their main parameters are approaching the requirements values, i.e., large number of actuators (up to 4096, see Figure 2), large stroke (up to 5.5 µm for lower actuator count devices), and good surface quality. 

BMC is currently conducting several actions for developing DMs devoted to space applications. In 2011, a classical DM has been tested with a Sounding Rocket in the US, in the Planetary Imaging Concept Testbed Using a Rocket Experiment (PICTURE). PICTURE-B has been launched in November 2015. This experiment measures directly optical light scattered by the debris disk around Epsilon-Eri star. In parallel, several studies have been engaged to modify the DM architecture and make it compatible with space environment [8].

### 2.2. Iris AO Deformable Mirror

Iris AO has been manufacturing piston-tilt-tilt (PTT) MEMS DMs with high-optical-quality mirror segments since 2002. University research that demonstrated the basic concept was first reported in 2001 [9] and early demonstrations of products were reported some years later [10]. Current products span apertures of 3.5 mm–7.7 mm with 111–489 actuators. A 939 actuator DM will be available in late 2017 and development is underway on a 3000 actuator PTT DM.

**Figure 3:**
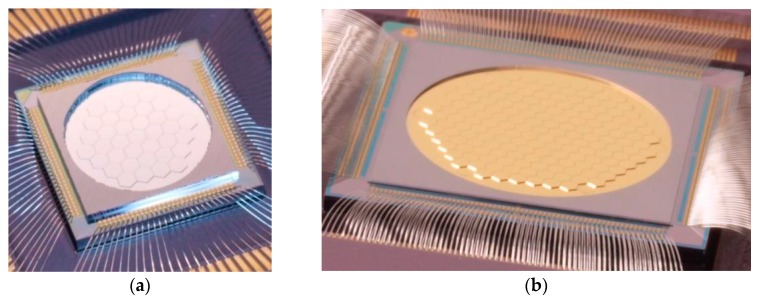
(**a**) Photograph of protected-silver coated PTT111 Deformable Mirror (DM) mounted in a ceramic package. The 111 actuator DM is comprised of 37 PTT segments tiled in a hex-packed array with an inscribed aperture of 3.5 mm; (**b**) Photograph of gold-coated PTT489 DM. The 489 actuator DM is comprised of 163 PTT segments with an inscribed aperture of 7.7 mm. The 700 µm vertex-to-vertex segment footprint for the PTT111 and PTT489 DM are identical.

Figure 3 shows photographs of two different segmented DMs manufactured by Iris AO with 111 (PTT111) and 489 (PTT489) actuators. The DM segments are independent and are controlled by three actuators to give three degrees of freedom. Thus, the PTT111 DM has a total of 37 independent PTT segments and the PTT489 DM has 163. The PTT111 DM shown here is coated with a protected-silver coating. The PTT489 DM is shown with a gold coating. The coatings are typical coatings used for macroscale optics. They are much thicker than typical coatings used on MEMS and thin-membrane mirrors, so they are very robust. This is possible because the segment design and fabrication technology is very tolerant to various coatings. Even high-reflectance dielectric coatings for wavelengths ranging from 255 nm–1600 nm are available as options.

**Figure 4:**
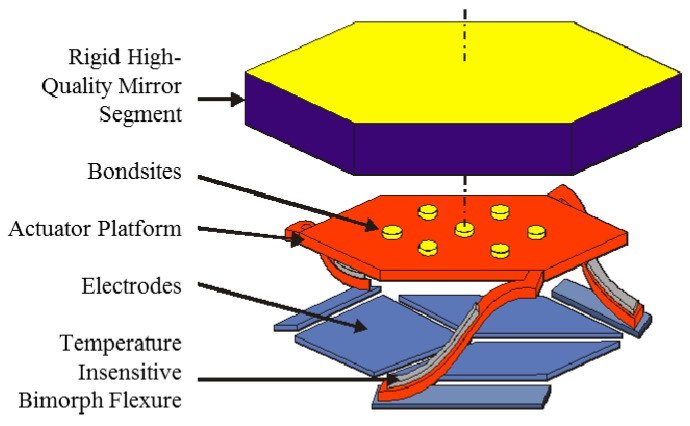
Exploded-view schematic diagram of a 700 µm circumscribed diameter (606.2 µm flat-to-flat pitch) mirror segment. Scaling is highly exaggerated in the vertical direction. Actuator platform heights are in the range of 20–40 µm for these segments, which enables 8 µm of stroke.

Figure 4 shows an exploded-view schematic diagram of one of the mirror segments. During manufacturing, the rigid mirror segments are bonded to actuator arrays at the chip level prior to the microstructure release step. After removing the sacrificial layer, the segments elevate above the underlying electrodes because of engineered residual stresses in the bimorph and actuator platform materials. The proprietary material stack is chosen to minimize elevation due to changes in temperature. The scaling in the vertical direction is highly exaggerated in Figure 4. In reality, the platform elevation is up to 40 µm whereas the segments are 350 µm on a side (700 µm across). The three underlying actuator electrodes enable piston-tip-tilt (PTT) positioning. Factory calibration and an open-loop controller conveniently convert from a desired position to actuator voltages [11].

The DM is manufactured using typical MEMS and integrated circuit materials such as polycrystalline silicon (polysilicon), silicon dioxides, silicon nitrides, and a proprietary bimorph material with similar coefficient of thermal expansion (CTE) to that of polysilicon. After the DM is fabricated using highly stable MEMS materials, it is mounted onto a ceramic pin-grid array (PGA) package using an epoxy. The DM is sealed in nitrogen by epoxying a cover window over the DM.

## 3. Cryogenic Interferometric Test Set-Up

Over the last few years, LAM has developed expertise in the characterization of micro-optical components, especially for small-scale deformation characterization on their surface from room temperature down to cryogenic temperatures. The aim is to be able to use these devices in all type of spectrographs, from ground-based to space telescopes as well as from Visible to IR instruments. The background of this test set-up development was in the framework of the NASA study of NIRSpec for the JWST.

### 3.1. Interferometric Bench

The measurement of the shape and the deformation parameters of the MOEMS devices is made thanks to an interferometric characterization bench, fully developed in-house (hardware and software). Characterizations are done in static or dynamic modes, including measurements of optical surface quality at different scales, actuator stroke, maximum mirror deformation, and cut-off frequency; the latest parameter has not yet been measured in this study.

This bench is a high-resolution and low-coherence Twyman-Green interferometer. It is a modular set-up around three main parts: illumination, interferometric cavity, and imaging system. *The illumination* is provided by a halogen lamp with an interference filter (typical example: λ_0_ = 650 nm, ∆λ = 10 nm). Fixing the temporal light coherence eliminates all parasitic fringe patterns induced by classical high coherence sources such as lasers. *The interferometric cavity* is clear of any optical components in order to keep the highest optical quality by avoiding any differential aberration introduced by these additional elements. *The imaging system* offers two magnification configurations by a simple lens change: (1) a high in-plane resolution or (2) a large field of view authorizing either a very sharp (around 2 µm) analysis of the micro-mirror structure inside a small field (typically 1 mm), or the whole device study with a larger size (up to 40 mm).

Out-of-plane measurements are performed with phase-shifting interferometry showing very high resolution (standard deviation < 1 nm). The bench is mounted on a damped optical table, and surrounded by a Plexiglas enclosure. Features such as optical quality or electro-mechanical behavior are extracted from these high precision three-dimensional component maps. Range is increased without loosing accuracy by using two-wavelength phase-shifting interferometry authorizing large steps measurements. Dynamic analysis like vibration mode and cut-off frequency could be also measured with time-averaged interferometry [12].

### 3.2. Cryogenic Experiment

**Figure 5:**
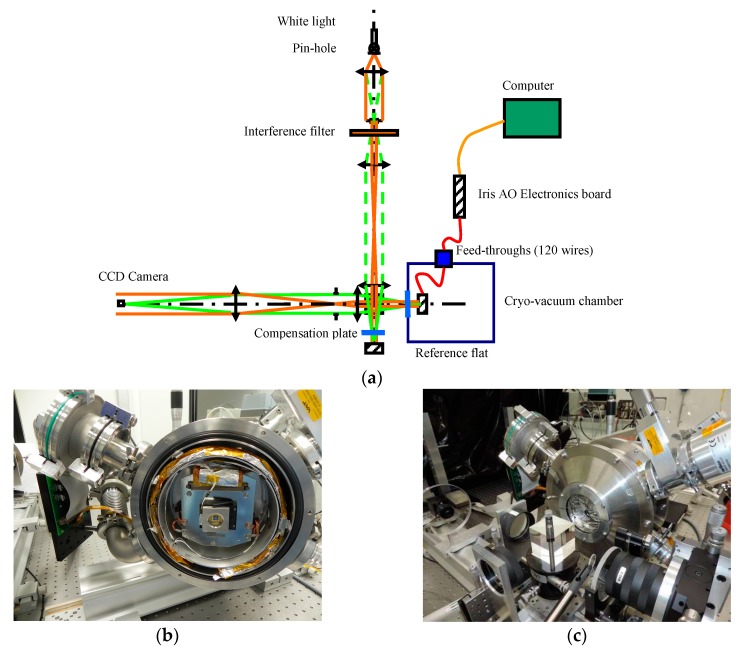
(**a**) Schematic description of our interferometric measurement set-up with the cryo-vacuum chamber; (**b**) PTT111 device mounted in the cryogenic chamber for characterization in a space environment; (**c**) Front window is closed and the cryogenic chamber is installed in front of our interferometric setup. The segmented deformable mirror could be successfully actuated before, during, and after cryogenic cooling at 160 K.

In front of our interferometric setup has been installed a cryo chamber for reaching temperatures as low as 100 K when not loaded, using a cryogenic generator, and vacuum pressure in the range of 10^−6^ mbar (Figure 5a). The chamber is equipped with an internal screen radiatively insulating the sample from the chamber. Temperature sensors and local heaters are used for controlling the environment, and connected to a custom built control electronics and control-command software. The component is monitored continuously thanks to a glass window at the entrance of the chamber. As we are using low coherence interferometry, the fringe contrast can be kept at maximum by balancing the optical path in the reference arm of the interferometer with a compensation plate identical to the chamber window. In our set-up, the chamber window and the compensation plate have been manufactured at the same time in the same glass material [13].

### 3.3. PTT111 Mounting

The PTT111 device is packaged in PGA chip carrier. The PGA is inserted in a ZIF-holder integrated on a PCB board. Large metallic surfaces on the PCB facilitate cooling down the system; eliminating the solder-stop layer eases outgassing of the PCB base material during evacuation of the chamber. The PCB itself is mounted via a fix-point-plane-plane attachment system to a solid aluminum block, the latter being interconnected to the cryo-generator (Figure 5b). Thick copper wires between the PCB and the aluminum block further enhance thermal transport between the sample chip and the cryostat. Two ribbon cables allow interconnecting up to 120 electrical wires through two 100-pin and 20-pin feed-throughs. Outside of the cryo chamber, the wires are connected to the Iris AO control electronics. Temperature sensors are connected to the aluminum block and to the window frame on top of the device.

The chamber is then closed by a flange and placed in front of the interferometer (Figure 5c). Along the reference path, two compensation plates are placed for compensating the chamber window (large plate) and the device window (small plate). By this way, we keep a high contrast for the interferometric fringes.

## 4. PTT111 Surface Characterization

**Figure 6:**
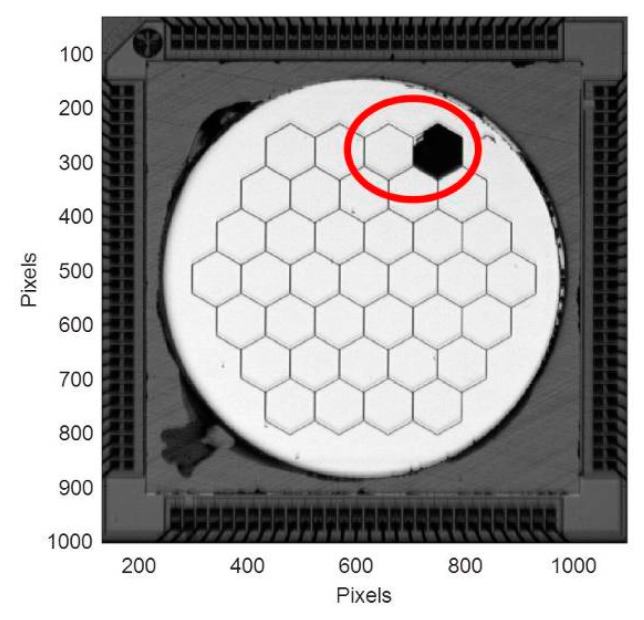
PTT111 device tested in our experiment (engineering grade device). Segments #23 and #24 (in the red circle) are lockouts.

These first experiments are done using an engineering grade device where segments #23 and #24 are inactive because of defects in the DM that occurred during manufacturing. The manufacturer calls these locked segments or lockouts as they are not controllable. Because the segments are independent, the defects only affect the locked segments and not neighboring one. The segment thickness is 25 µm and the coating is protected silver. The maximum array stroke after flattening is 3.01 µm, and the maximum tilt angle is 5 mrad. Figure 6 is a picture of the device made on our bench (without the interferometric fringes). The two lockouts segments are at the upper right.

The device is driven by the *Graphical User Interface* (GUI) provided by Iris AO for the integration and pre-characterization phases. The interferometric measurements are done with the LAM-developed software, in Matlab, and linked with the Matlab driver provided by Iris AO. The GUI is showing a view of the mirror with numbered segments, and the global Zernike coefficient as well as local (at segment level) piston/tip/tilt positions could be tuned for each segment.

**Figure 7:**
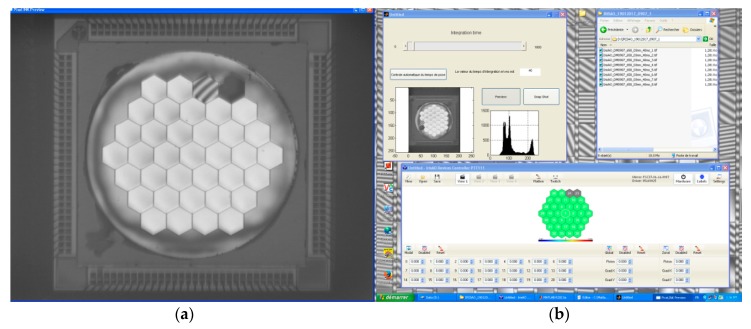
Screen shot during the experiment; (**a**) The interferometric image of the mirror when the “best flat” condition is applied; (**b**) The Iris AO GUI for driving the device.

A calibration step has been done by Iris AO for measuring each actuator response (3 actuators/segment). Then, a “best flat” condition is calculated in order to minimize the residual wavefront error on the surface, and applied. Figure 7 is a screen shot made at the beginning of the experiment when the best flat condition is applied; at the left hand side is the interferometric image of the mirror with the “best flat” condition; at the right bottom is the view of the Iris AO GUI for driving the device.

**Figure 8:**
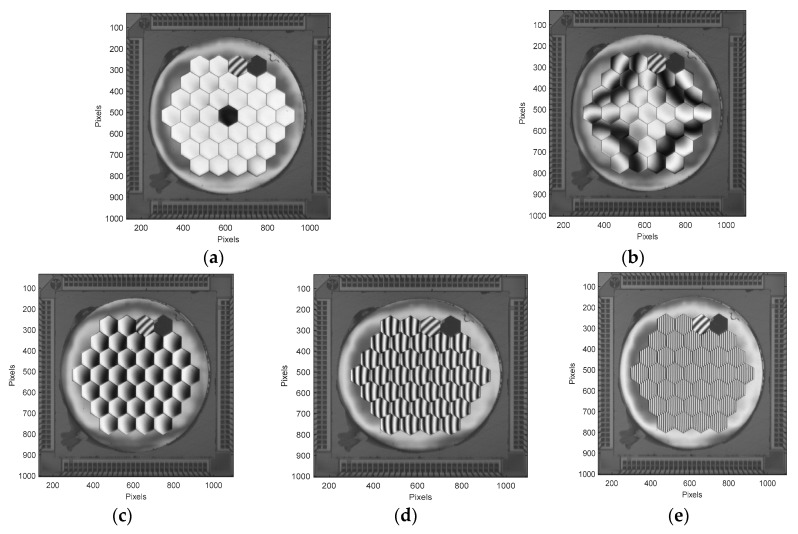
(**a**) 150 nm piston on segment#1; (**b**) astigmatism (0.125 on Zernike coefficient); (**c**–**e**) Increasing tilt values along the X direction are applied to all segments (0.25, 1.5 and 4.9 mrad).

We could then apply a series of commands to the mirror. In Figure 8, different mirror configurations are presented. From top left to bottom right, we can see:

- a pure piston (150 nm) on the central M1 segment,
- a global astigmatism on the mirror, using the global Zernike coefficient set at 0.125,
- a series of three identical tilts on all segments in X direction, with 0.25 mrad, 1.5 mrad, and 4.9 mrad respectively.

### 4.1. Best Flat at Ambient

**Figure 9:**
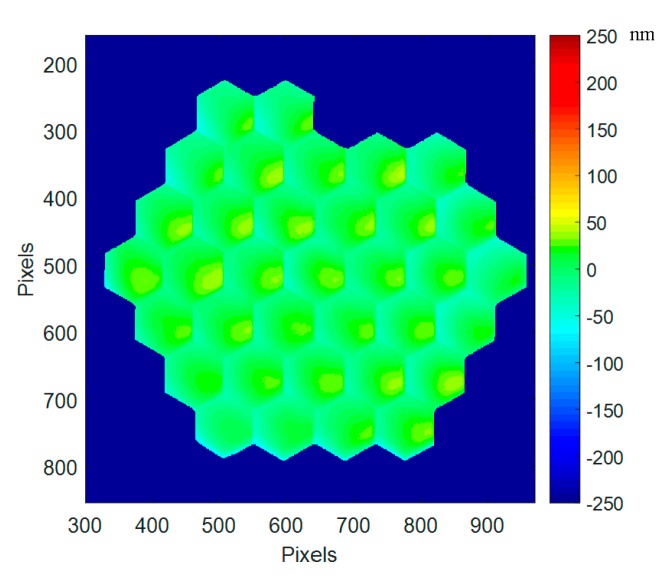
Best flat surface deformation at ambient (17 nm RMS, 123 nm PtV) when the best flat condition calibrated by Iris AO is applied; the gravity effect has not been removed.

From the interferometric measurement, we obtain the surface deformation of the deformable mirror; in the whole paper, deformation values are always given in nanometers, and maps are displayed with a ±250 nm range for better comparison between related Figure 9, 11, and 13a–15a. 

The best flat residual over the whole mirror is very good with 17 nm RMS, 123 nm PtV (Figure 9). Note that this best flat provided by Iris AO is not corrected from the gravity effect occurring in our experiment, as the PTT111 is mounted in vertical position.

This result shows the high quality of the mirror architecture and of the fabrication process. This flatness is a combination of a very good reproducibility of the actuator platform position after his elevation thanks to the bimorph flexures (Figure 4), and the choice of thick single-crystalline Silicon for the segment material. This position is very stable; long term measurement has been done at ambient on position stability [6] and reproducibility, but this has not been done yet in cryo.

At segment level, the residual wavefront error is with a slight convex shape, observed on most of the segments (see Section 4.6).

**Figure 10:**
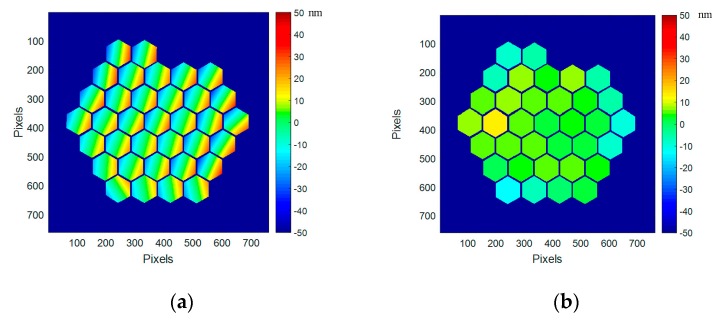
Residual tip-tilts (**a**) and pistons (**b**) at ambient, when the best flat condition from Iris AO is applied; the gravity effect has not been removed.

We measure the mirror surface shape with high accuracy and sort out tilt and piston values for wach segment. They are displayed on segment maps in Figure 10. Over the maps, the tilts have a value of 32 µrad RMS (140 µrad PtV) while the pistons expands on 6.2 nm RMS (28 nm PtV). The residual tilts are mainly due to the gravity effect as the device is mounted in vertical position in our measurement set-up.

### 4.2. Best Flat at 160 K, First Run

The device is then cooled down slowly from ambient temperature (293 K) down to 160 K, with the device constantly operating in its best flat condition. The PTT111 device is operating properly at all temperatures between 293 K and 160 K, and in vacuum.

Every 10 K an interferometric measurement is done in order to follow the differential deformation of the mirror at whole mirror level as well as at segment level. Several patterns are applied and measured in order to see the ability of the device to behave as at room temperature; the applied “patterns” are best flat, pure pistons on some segments, and different tilts on the segments. Due to the vibrations induced by the cryo pump on the sample, we have to stop it during the measurement, leading to a limited increase of the temperature during the measurement duration. Phase shifting interferometry parameters have been adjusted in order to minimize the measurement time to a few minutes.

**Figure 11:**
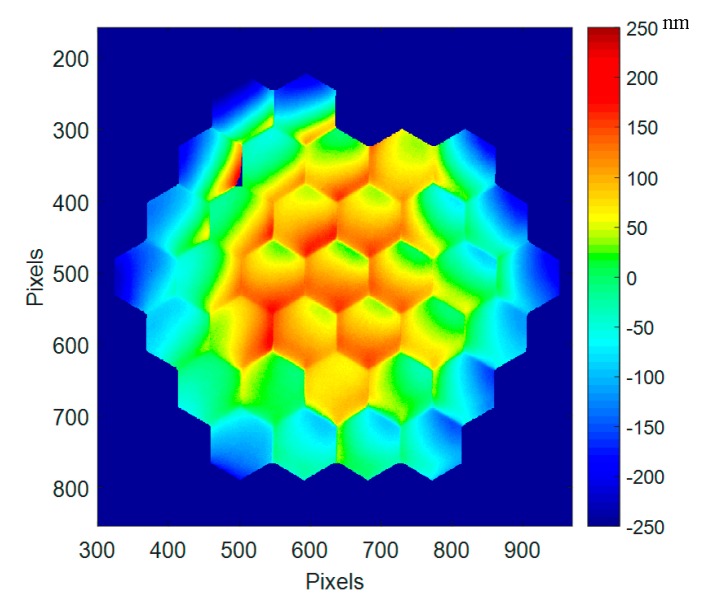
Best flat mirror deformation at cryo (160 K), first run, with the original best flat calibrated at ambient (86 nm RMS, 501 nm PtV).

In Figure 11, the best flat surface deformation at cryo (160 K) is given with the original best flat condition as calibrated at ambient. A global convex deformation is observed reaching a deformation of 86 nm RMS, 501 nm PtV. Some additional deformations (mainly tilts) are observed on some segments at the upper left side (segments #26, 27, and 28).

The global convex shape in cryo is due to the packaging “shrinking” in cryo. The Coefficient of Thermal Expansion (CTE) mismatch between die/package materials induces a global effect on the mirror when cooled down at 160 K. 

The mirror is operating perfectly in cryo, and a “new” best flat condition will be developed and described in the next paragraphs. 

**Figure 12:**
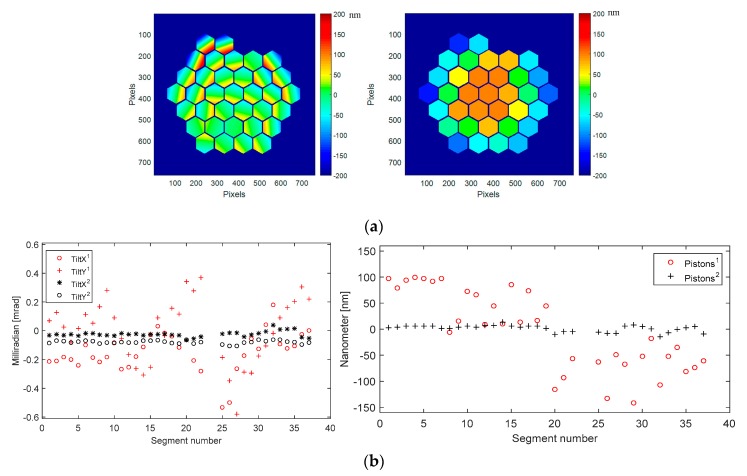
(**a**) Segment maps of the residual tip-tilts and pistons at cryo (160 K), first run; (**b**) Values for each of the 37 segments of PTT111 (in red tilt X and tilt Y at cryo, in black, for reference, values at ambient). Residual tilts at ambient (black signs) are 32 µrad RMS (140 µrad PtV), and 200 µrad RMS (950 µrad PTV) in cryo (red signs). Residual pistons at ambient (black signs) are 6.2 nm RMS (28 nm PtV), and 74 nm RMS (239 nm PTV) in cryo (red signs).

We measure and sort out for each segment tilt and piston values. They are displayed on segment maps and shown in graphs, in Figure 12. The tilts have a value of 200 µrad RMS (950 µrad PtV) while the pistons expands on 74 nm RMS (239 nm PtV). Tilts and pistons are not behaving the same way. Pistons are behaving within the three concentric rings of PTT111 37 segments, with a common motion for the central area (segments #1 to 7), the middle ring (segments #8 to 19), and the outer ring (segments #20 to 37); in Figure 12, residual pistons at ambient (black signs) are 6.2 nm RMS (28 nm PtV), and 74 nm RMS (239 nm PTV) in cryo (red signs); this effect is clearly related to the shrinkage of the overall device. As for the tilts, they don’t show a clear pattern; they are scattered away from the original positions at ambient. In Figure 12, residual tilts at ambient (black signs) are 32 µrad RMS (140 µrad PtV), and 200 µrad RMS (950 µrad PTV) in cryo (red signs); this differential evolution is possibly due to the different modification of the complex structure underneath each segment: the actuator platform, the bonding pads, the mirror segment, the coating, and the underlying 3 legs bimorph structure.

These effects will be corrected in a second step (see Section 4.4).

### 4.3. Best Flat at Ambient (Back 1)

**Figure 13:**
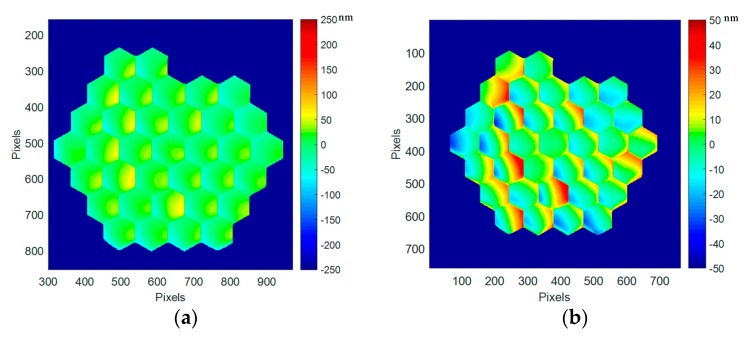
Mirror surface deformation at ambient (293 K) when best flat Iris AO condition is applied, after one cooling cycle: (**a**) Surface deformation map (22 nm RMS, 123 nm PtV); (**b**) Difference between the two surface deformation maps before and after one cooling cycle.

When the device is warmed up back to ambient temperature we measure its surface shape in the original best flat mode again, and we get a slightly different result, as revealed in Figure 13.

The degradation of the surface deformation is going up from 17 nm to 22 nm RMS (123 nm to 152 nm PtV), the differences being mainly due to the additional tilts on each segment by 0.15 mrad PtV over the device, while the pistons stay within the same range as before the cooling cycle. This is probably due to the stress relaxation process in the complex structure supporting each segment, leading to a slightly different evolution from segment to segment. As for the segment mirror surface, there is no difference (below 1 nm), thanks to the high quality of the segment made of single-crystalline silicon material. This effect could be described as an annealing process.

**Figure 14:**
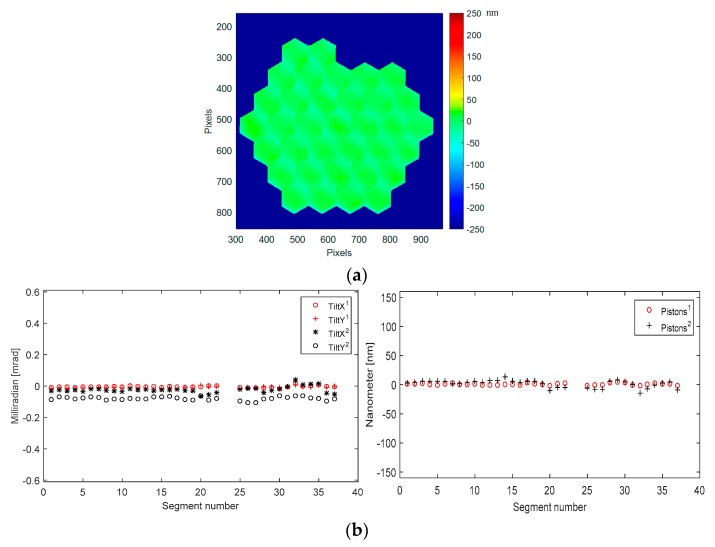
Mirror surface deformation at ambient (293 K) when *improved best flat* condition is applied, after one cycle of cooling: (**a**) Surface deformation map (10 nm RMS, 79 nm PtV); (**b**) Residual tilts and pistons values for each of the 37 segments of PTT111. Residual tilts with improved best flat (red signs) are 6.5 µrad RMS (36 µrad PtV), while they were 32 µrad RMS (140 µrad PTV) with the original best flat (black signs). Residual pistons with improved best flat (red signs) are 1.6 nm RMS (7 nm PtV), while they were 6.2 nm RMS (28 nm PtV) with the original best flat (black signs).

In order to improve the best flat quality, we decided to develop an improved best flat procedure by measuring, with high accuracy, the tip-tilt and piston residuals and combining them with the original best flat values calibrated by Iris AO. We then obtain the *improved best flat* shown in Figure 14. The mirror surface deformation is then as low as 10 nm RMS, 79 nm PtV.

In the graphs of Figure 14, residual tilts with improved best flat (red signs) are 6.5 µrad RMS (36 µrad PtV), while they were 32 µrad RMS (140 µrad PTV) with the original best flat (black signs). Residual pistons with improved best flat (red signs) are 1.6 nm RMS (7 nm PtV), while they were 6.2 nm RMS (28 nm PtV) with the original best flat (black signs).

*Improved best flat* condition will be used in the following experiments. 

### 4.4. Best Flat at 160 K, Second Run

In order to demonstrate full operation of PTT111 at cryogenic temperature, we decide to cool down the device and optimize in-situ all actuators for generating a cryo best flat.

**Figure 15:**
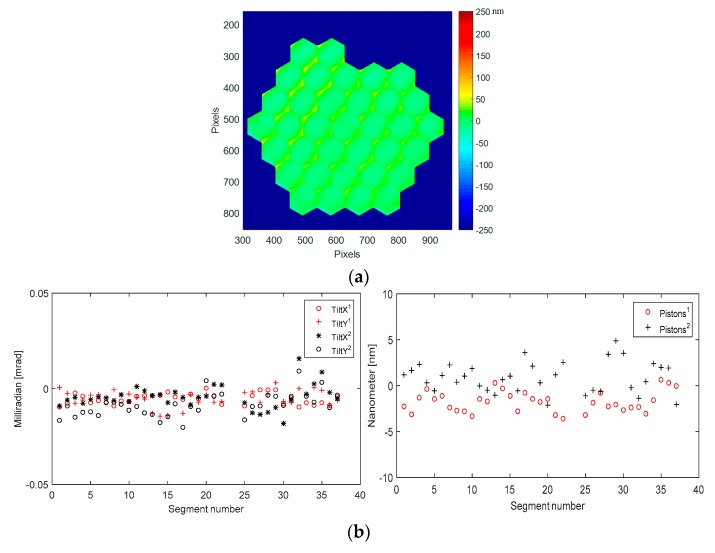
Mirror surface deformation at cryo (160 K), second run, when *cryo best flat* condition is applied: (**a**) Surface deformation map (12 nm RMS, 113 nm PtV); (**b**) Residual tilts and pistons values for each of the 37 segments of PTT111. Residual tilts with *cryo best flat* (red signs) are 3.5 µrad RMS (17 µrad PtV), while they were 6.5 µrad RMS (36 µrad PtV) with the *improved best flat* at ambient (black signs). Residual pistons with *cryo best flat* (red signs) are 1.2 nm rms (4.3 nm PtV), while they were 1.6 nm RMS (7 nm PtV) with the *improved best flat* at ambient (black signs).

Our strategy is a weighted addition of the consecutive measurement residual errors and, using Iris AO electronics, we are loading these calculated values actuator by actuator, departing from the original values provided by Iris AO, and applyling them to the device. Our *cryo best flat* condition is a combination of [best flat calibrated by Iris-AO at ambient, improved best flat at ambient, improved best flat in cryo (first run), improved best flat in cryo (second run)]. Then, in a single measurement step and applying this best flat condition, we got, at 160 K, a mirror surface deformation as low as 12 nm RMS, 113 nm PtV (Figure 15).

Our *cryo best flat* at 160 K is then very close to our *improved best flat* at ambient (293 K), showing our ability to operate properly PTT111 device in cryo. The deformation difference is 2 nm RMS, 34 nm PtV between 160 K and 293 K. This additional deformation is due mainly to the mirror segment deformation, as revealed in the following Section 4.6.

In the graphs of Figure 15, residual tilts with *cryo best flat* (red signs) are 3.5 µrad RMS (17 µrad PtV), while they were 6.5 µrad RMS (36 µrad PtV) with the *improved best flat* at ambient (black signs). Residual pistons with *cryo best flat* (red signs) are 1.2 nm rms (4.3 nm PtV), while they were 1.6 nm RMS (7 nm PtV) with the *improved best flat* at ambient (black signs).

A second loop of best flat optimisation is useless as the remaining mirror surface deformation is only due to the contributions of individual segment deformations; this is clearly visible in the surface deformation map of Figure 15.

The additional deformation of PTT111 at cryo is 501 nm PtV (Figure 11). The *cryo best flat* is compensating this deformation, minimizing the whole mirror deformation, down to 12 nm RMS (123 nm PtV). The maximum stroke of this device being 3.01 µm at ambient, then the operational stroke is reduced to 2.5 µm at cryo (16.7% stroke reduction).

### 4.5. Best Flat at Ambient (Back 2)

When the device is warmed up back to ambient temperature we measure its surface shape in the improved best flat mode again, and we get nearly exactly the same surface, as shown in Figure 14. The device seems to now be stabilized with no evolution after a complete cycle at cryogenic temperature.

A thermal cycling test might be useful to confirm this result.

### 4.6. Analysis at Segment Level

**Figure 16:**
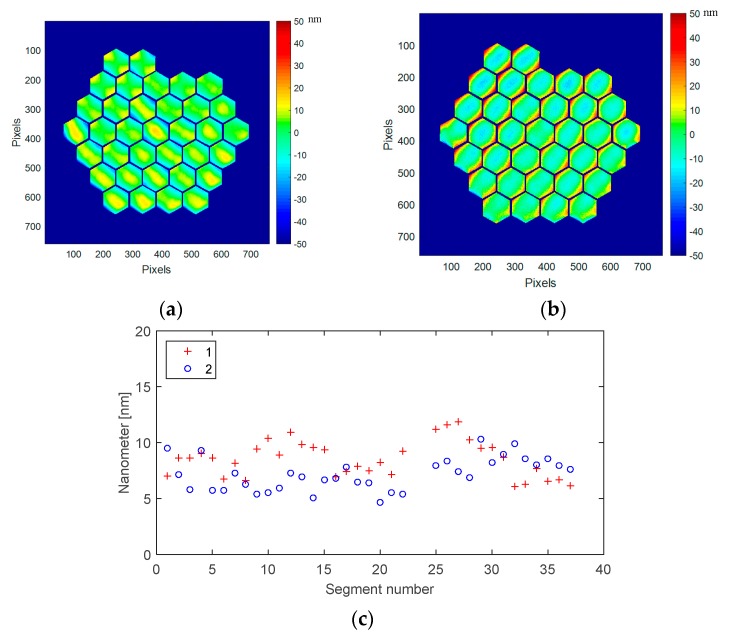
DM segments surface deformation: (**a**) At ambient (293 K), 5 nm RMS, 24 nm PtV; (**b**) At 160 K 8 nm RMS, 47 nm PtV; (**c**) Surface deformation by segments at ambient (values 2 in blue) has a mean value of 7.2 nm with a standard deviation of 1.5 nm, and at 160 K (values 1 in red), a mean value of 8.5 nm with a standard deviation of 1.6 nm.

Thanks to our set-up spatial resolution, we have several thousand measurement points per segment. It is then possible to measure, at segment level, the deformation induced by the strong temperature change from ambient to cryo. Figure 16a,b are identical to Figure 14a and Figure 15a, with the mirror segment marked, and an expended scale for revealing the local effects on each segment. The surface deformation by segments at ambient (values 2 in blue) has a mean value of 7.2 nm with a standard deviation of 1.5 nm, and at 160 K (values 1 in red), a mean value of 8.5 nm with a standard deviation of 1.6 nm.

**Figure 17:**
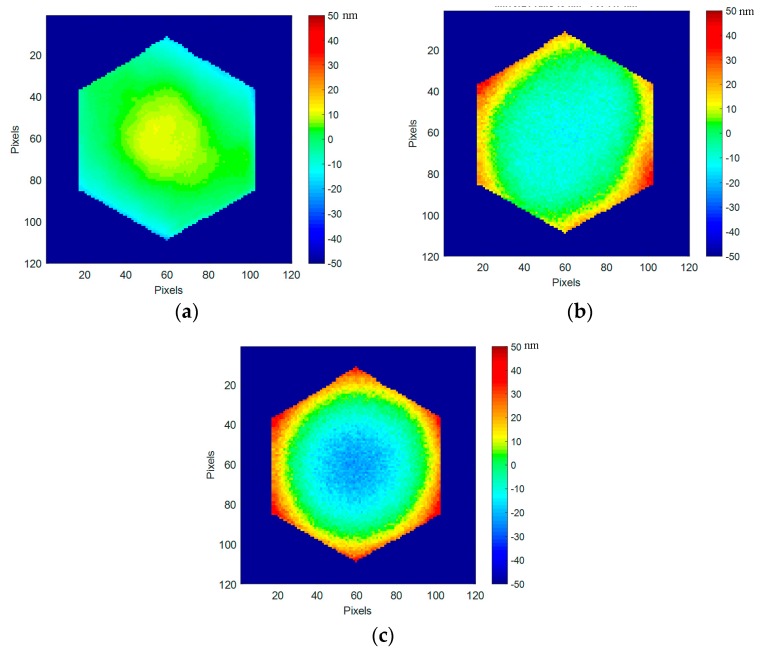
DM segment #21 surface deformation: (**a**) At ambient (293 K), 5 nm RMS, 24 nm PtV; (**b**) At 160 K 8 nm RMS, 47 nm PtV; (**c**) Deformation difference between ambient and 160 K, 4.9 nm RMS, 71 nm PtV.

By selecting a typical segment, a closer analysis of the segment evolution at cryogenic temperature could be done, especially on its shape. In Figure 17, on segment #21, we can clearly see that the convex cylindrical shape at ambient is changing to an astigmatic concave shape at cryo. At ambient (293 K), the segment surface deformation is 5 nm RMS (24 nm PtV), while at 160 K the deformation is still low, at 8 nm RMS (47 nm PtV). 

**Figure 18:**
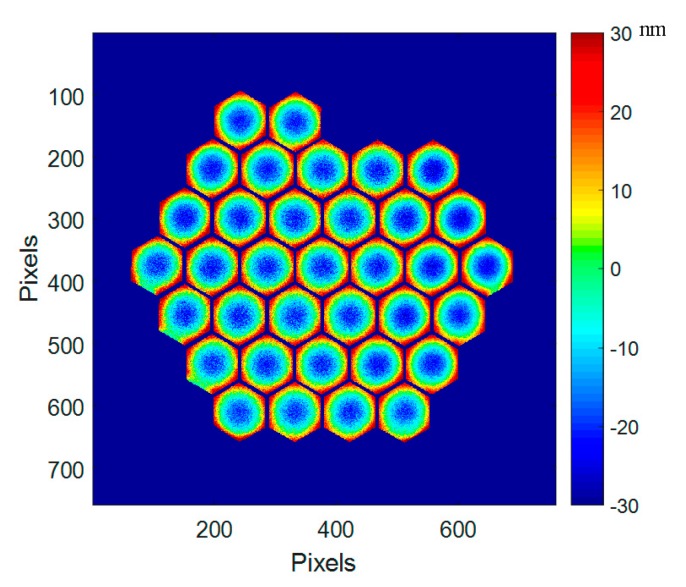
Segment deformation induced by the temperature change between ambient and cryo (160 K); map of all segments of PTT111.

A very interesting feature is observed when looking at the deformation difference between ambient and 160 K (Figure 17c): it reveals a pure concave axisymetrical change of 4.9 nm RMS (71 nm PtV). This is due to the CTE mismatch between the single-crystalline silicon and the silver-protected coating deposited on top of the segment. All segments are behaving in the same way as shown in Figure 18. 

The mean deformation at ambient is in the range of 25 nm, while it rises to 50 nm in cryo. This deformation difference is still within the requirement of almost all foreseen wavefront correction systems. This deformation difference at segment level is the major contribution to the whole mirror surface deformation, as described in Section 4.4.

## 5. Conclusions

Innovative wavefront correction systems in existing telescopes on the ground and in space, as well as in the future ELTs, need efficient MOEMS Deformable Mirrors (DM) able to perform at room temperature as well as in cryogenic and vacuum environments.

Using a specific interferometric bench coupled with a cryo-vacuum chamber, a PTT 111 DM from Iris AO has been successfully tested from ambient temperature to 160 K. The device is properly operating in cryo, revealing an additional, mainly focus-like, 500 nm deformation at 160 K; we were able to recover the best flat in cryo by correcting the focus and local tip-tilts on all segments, reaching 12 nm RMS on the entire surface. 

Tests on DMs with different mirror thicknesses (25 µm and 50 µm) and different coatings (silver and gold) are currently under way.

Finally, the goal of these studies is to test DMs in cryo and vacuum conditions as well as to improve their architecture for stable operation in a harsh environment.
